# Wearable Healthcare Monitoring Based on a Microfluidic Electrochemical Integrated Device for Sensing Glucose in Natural Sweat

**DOI:** 10.3390/s22228971

**Published:** 2022-11-19

**Authors:** Zouaghi Noura, Imran Shah, Shahid Aziz, Aamouche Ahmed, Dong-Won Jung, Lakssir Brahim, Ressami ElMostafa

**Affiliations:** 1 National School of Applied Sciences, LISA Laboratory, Cadi Ayyad University, Marrakech 40000, Morocco; 2 Moroccan Foundation for Advanced Science, Innovation and Research, Digitalization & Microelectronics Smart Devices Laboratory, Rabat 10100, Morocco; 3 Department of Aerospace Engineering, College of Aeronautical Engineering, National University of Sciences and Technology, Risalpur 24090, Pakistan; 4 Department of Mechanical Engineering, Jeju National University, 102 Jejudaehak-ro, Jeju-si 63243, Republic of Korea

**Keywords:** sweat, wearable microfluidic sensors, screen-printed electrode, glucose detection

## Abstract

Wearable sweat sensors offer the possibility of continuous real-time health monitoring of an individual at a low cost without invasion. A variety of sweat glucose sensors have been developed thus far to help diabetes patients frequently monitor blood glucose levels through sweat glucose as a surrogate marker. The present study demonstrates the development and characterization of a three-dimensional paper-based microfluidic electrochemical integrated device (3D PMED) for measuring glucose concentration in sweat in real-time via simple, non-invasive, capillary-action-based sample collection. The device was selective for glucose, and it detected glucose accurately in the clinically relevant range (0~2 mM) in an off-body setup. To the best of our knowledge, this is the first time NEXAR™ has been used for biosensing applications. Further, the developed glucose sensor has acceptable sensitivity of 16.8 µA/mM/cm^2^. Importantly, in an on-body setup, the device achieved a significant amperometric response to sweat glucose in a very short amount of time (a few seconds). With detailed investigations, this proof-of-concept study could help further the development of sensitive and selective sweat-based glucose sensing devices for real-time glucose monitoring in diabetes patients.

## 1. Introduction

Wearable microfluidic sensors form a rapidly evolving field of research in biomedical engineering. They offer several advantages such as low cost, the ability to track an individual’s health status continuously (or semi-continuously) in real-time, non-invasive (or minimally invasive) testing of analytes and health parameters, and at-home and on-body testing [1,2].

Sweat sensors are among the most commonly developed and studied wearable sensors [1,3,4,5,6,7]. Sweat contains a wide range of metabolites that carry important information regarding human health, such as glucose (diabetes marker), lactic acid (local ischemia marker), proteins and amino acids (schizophrenia, atopic dermatitis, etc.), and chloride (cystic fibrosis marker) [4,8]. Therefore, wearable sweat sensors offer the possibility of continuous real-time health monitoring of an individual at a low cost and with no invasion.

A variety of natural sweat glucose sensors have been studied thus far to help diabetes patients frequently monitor blood glucose levels through sweat glucose as a surrogate marker [9,10,11,12]. These sensors use both enzymatic and non-enzymatic detection technologies and offer a wide range of acceptable glucose detection sensitivities, from 22 µA/mM/cm^2^ to 230 µA/mM/cm^2^ [9,11,12,13,14,15]. Some of these sensors use the fingertip as the sample collection surface, owing to its high sweat rate [10], while others use wrists, arms, or the forehead [14].

However, sweat sensors are associated with numerous unaddressed challenges. These include issues with sweat collection, the impact of external environmental factors on sweat production rate, and handling battery requirements for continuous electrolyte monitoring [7]. Particularly with respect to glucose detection in sweat, current challenges include reproducibility of the sweat-based biosensor, selectivity issues due to interference from other sweat components, and establishing a correlation between sweat glucose measured by the wearable device and blood glucose measured through conventional analytical methods [2].

Our research demonstrates the development and characterization of a highly selective 3D PMED paper-based microfluidic electrochemical integrated device for measuring glucose concentration in natural sweat in real-time via simple, non-invasive, capillary-action-based sample collection. Importantly, NEXAR™ (Sigma-Aldrich, Shanghai, China) was used in place of the more commonly used Nafion^®^ membrane (Sigma-Aldrich, Shanghai, China)to improve the electrolyte separation capacity of the biosensor and thus reduce interference and improve selectivity. Both off-body and on-body tests of the fabricated device were performed in this proof-of-concept study.

## 2. Materials

The following reagents were used: glucose oxidase (Gox; 10 mg/mL) was obtained from Sigma-Aldrich (Shanghai, China). Phosphate-buffered saline (PBS; 0.01 M) was obtained from Sangon Biotech (Shanghai, China) Co., Ltd., and chitosan (5% *w*/*v*) and acetic acid (3% *v*/*v*) were purchased from Aladdin Ltd. (Shanghai, China) Sodium chloride, glucose (0–1.9 mM; 1 M), and NEXAR™ were obtained from Sigma-Aldrich(Shanghai, China).

For device fabrication, an electrochemical sensor was developed from screen-printed carbon electrodes (SPCEs) purchased from PalmSens B.V. (Randhoeve, The Netherlands). The SPCEs used in the present study had a conventional three-electrode configuration, with carbon as the working electrode (WE), silver as the reference electrode (RE), and carbon as the counter electrode (auxiliary electrode). Additionally, a screen mesh, wax (from crayons), and Whatman filter paper (cellulose paper) were used. Amperometric measurements were obtained using the PalmSens3™ potentiostat (PalmSens B.V., Randhoeve, The Netherlands), controlled by PSTrace software version number 5.8, PalmSens B.V. (Randhoeve, The Netherlands). All measurements were carried out at room temperature unless specified otherwise.

### 2.1. Electrode Surface Modification

**Figure 1:**
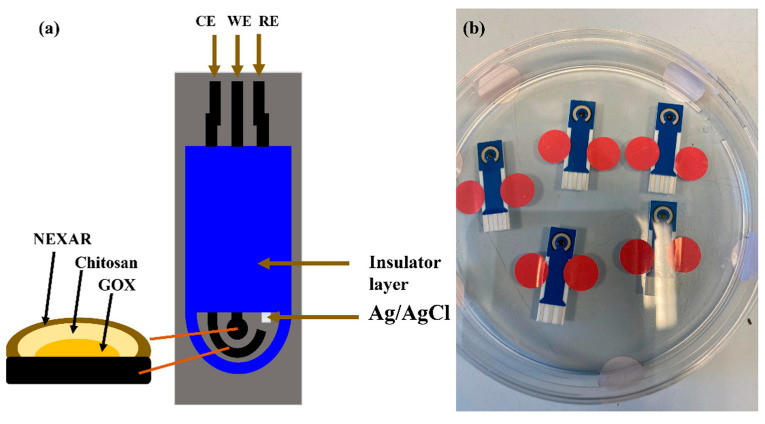
Characterization of the glucose sensor. (**a**). Schematic diagram of the glucose sensor. (**b**). Glucose-sensing electrode, coated with five layers.

The surface of the SPE used in the present study had a conventional three-electrode configuration, with carbon as the working electrode (WE), silver as the reference electrode (RE), and carbon as the counter electrode (auxiliary electrode) [16]. This three-electrode configuration was built on a corundum ceramic base (25.4 mm × 7.26 mm) and was modified through a five-step modification protocol. Firstly, 2 µL of a NEXAR™-ethanol mixture (1:10 *v*/*v*) were drop-casted on the working electrode’s surface. NEXAR™ helps separate interfering electroactive agents in sweat. The coating was allowed to air-dry for 30 min at constant room temperature. Thereafter, 3 µL of a chitosan solution (5% *w*/*v* in 3% *v*/*v* acetic acid) were cast on the electrode and subsequently left overnight (8 h) at 4 °C. Subsequently, 3 µL of GOx (10 mg/mL) were drop-casted on the surface of the working electrode, and the electrode was allowed to dry for 2 h at room temperature. Note that GOx acts as the catalyst for the conversion of glucose to gluconolactone, releasing hydrogen peroxide (H_2_O_2_) in the process. Electrochemical reduction results in the subsequent amperometric response recorded in this study. In the fourth step, 3 µL of a chitosan solution (5% *w*/*v* in 3% *v*/*v* acetic acid) were dropped on the electrode and allowed to dry at room temperature. Lastly, 2 µL of the Nexar™-ethanol mixture (1:10 *v*/*v*) were dropped on the working electrode, allowed to dry, and then stored at 4 °C until further use (Figure 1).

### 2.2. Device Fabrication

**Figure 2:**
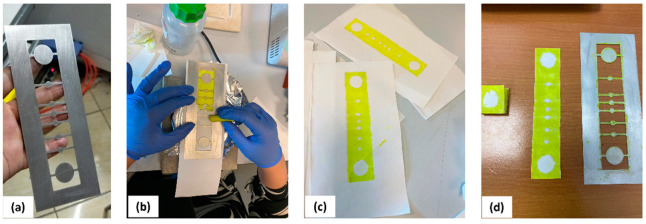
Fabrication process for 3D-PMED. (**a**,**b**) Coating cellulose paper with wax (crayon) on a heated surface, so only the exposed areas remain hydrophilic; (**c**,**d**) 3D PMED with sweat collector on one end and sweat evaporator on the other (both large).

Whatman qualitative cellulose filter paper was glued to the back of the screen mesh to prevent the paper from sliding during the process of screen printing. The screen mesh was rubbed and consequently heated (at 50–55 °C). Distinct patterns, as shown in Figure 2b,c, were created on the cellulose paper using wax crayons. The paper was then cut along the edge of the patterns (Figure 2c,d). Finally, the paper was folded four times as per the pattern to form five layers, the large, circular sweat collector, the vertical channel and transverse channels (formed by the smaller circles in between), the layer where the electrode will be attached, and the sweat evaporator layer (formed by the second large circle) (Figure 2a,d). The areas not coated with hydrophobic wax were designed to allow for the absorption of fluids and their capillary-action-based transfer to the evaporator end of the paper microfluidic device. Finally, the surface-modified electrode was introduced in the paper microfluidic setup to develop the glucose-sensing 3D PMED.

### 2.3. Off-Body and On-Body Characterization of the Glucose Sensor and the Paper-Based Microfluidic

The glucose sensor (electrode) was tested in an off-body setup by adding increasing concentrations of glucose (steps of 0.1 mM, range 0–1.6 mM glucose in 0.01 M PBS) onto the electrode surface. A calibration curve was generated based on the recorded amperometric response of the electrode. Thereafter, the integrated paper-based microfluidic was tested by adding glucose onto the collector end of the 3D PMED in 0.5 mM increments at intervals of 20 s. This experiment was used to measure the sensitivity of the device.

Finally, on-body testing of the paper-based microfluidic was performed. Briefly, a study participant was enrolled after the necessary information regarding the research study was provided to them. This was carried out in accordance with the “Moroccan Foundation for Advanced Science, Innovation and Research (MAScIR)” Ethical Committee. For the study, the paper-based microfluidic was securely placed on the forearm of the study participant, who was then asked to engage in physical exercise (cycling). The amperometric response of the device based on real-time sampling of sweat glucose was recorded and subsequently plotted.

## 3. Results

### 3.1. Field-Emission Scanning Electron Microscopy (FESEM) Analysis

**Figure 3:**
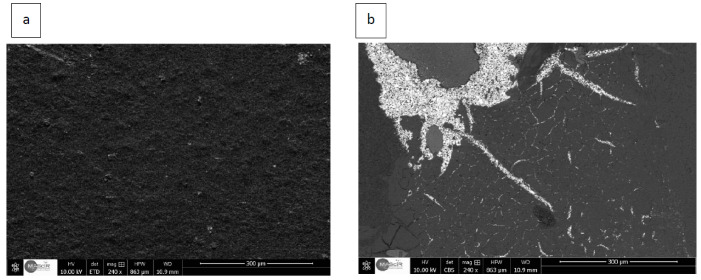
FESEM images of (**a**) bare SPCE and (**b**) SPCE surface modification.

The FESEM images of the screen-printed carbon electrodes at different preparation stages are displayed in Figure 3. It can be seen from the figure that the surface of modified SPCE has different morphology as compared to the bare electrode with spherical grains distributed among the carbon particles in the form of threads.

### 3.2. Off-Body Testing of the Glucose Sensor

**Figure 4:**
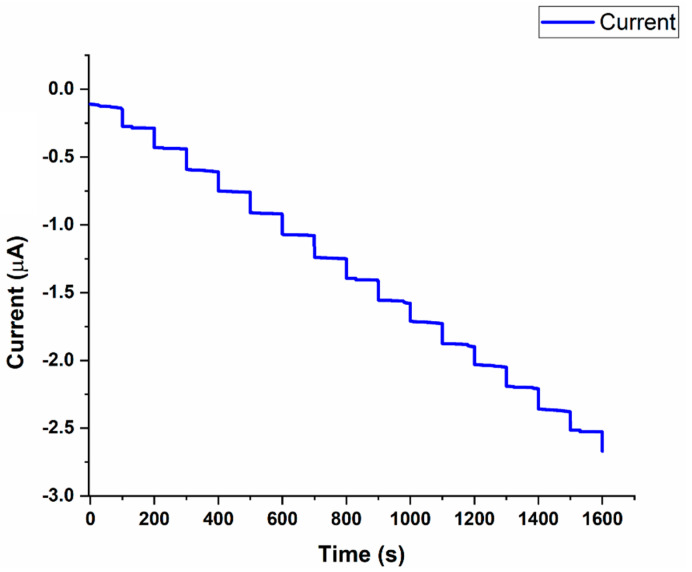
Amperometric current response at −0.1 V (w.r.t the Ag/AgCl reference electrode) with successive addition of 100 µM glucose concentration (0 µM~1.6 mM in 0.01 M PBS solution). Inset: Experimental setup used to develop the calibration plot.

Our developed glucose sensor was first used in an off-body setup to confirm its ability to detect glucose in simulated sweat (0.01 M PBS solution). A significant, reproducible, and sustained amperometric response was detected upon the successive addition of 0.1 mM glucose (in 0.01 M PBS) onto the electrode surface (Figure 4).

**Figure 5:**
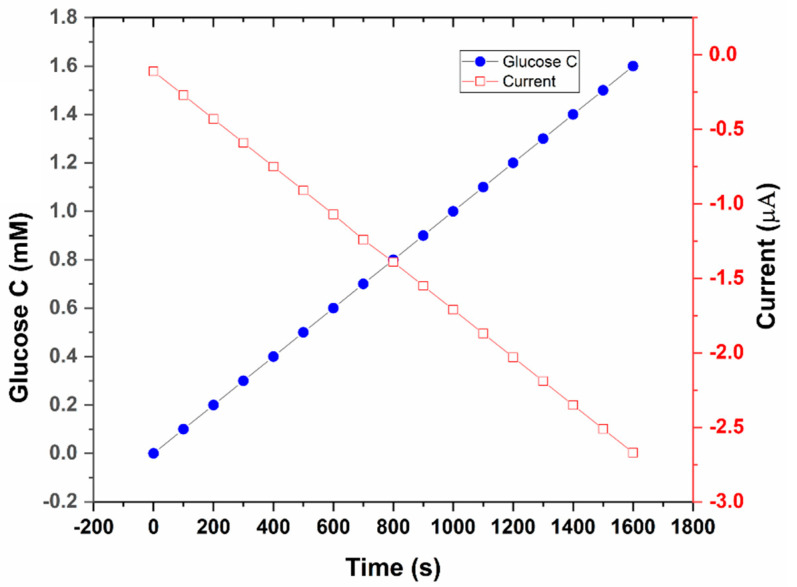
The linearity of the amperometric response of the glucose sensor at −0.1 V (w.r.t the Ag/AgCl reference electrode) at different glucose concentrations (0 mM~1.6 mM, in 0.01 M PBS solution).

With stepwise increases in glucose concentration, the current response also increased, as can be seen in Figure 4. The amperometric response of the glucose sensor was linear, with a coefficient of determination (R^2^ = 1), as shown in Figure 5.

### 3.3. Integrating the Glucose Sensor with the Paper-Based Microfluidic

**Figure 6:**
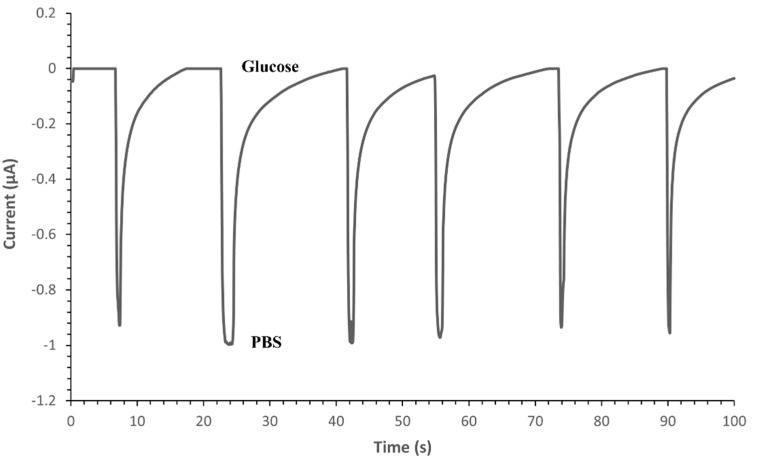
Amperometric current response at –0.1 V (vs. Ag/AgCl R.E.) with alternate injecting of glucose (0.5 mM~2.0 mM glucose in increments of 0.5 mM) and PBS.

After testing the glucose sensor independently, it was integrated with the 3D PMED. Subsequently, glucose solution was added to the collector of the 3D PMED in 0.5 mM increments (successively in 20 s intervals). The highest amperometric differential (0.42 µA) was noted upon the first addition of 0.5 mM glucose (Table 1 and Figure 6). Thus, the sensitivity of the 3D PMED glucose sensor was obtained as 0.42 µA/0.5 mM/0.05 cm^2^ (approximate electrode surface area), which equals 16.8 µA/mM/cm^2^.

**Figure 7:**
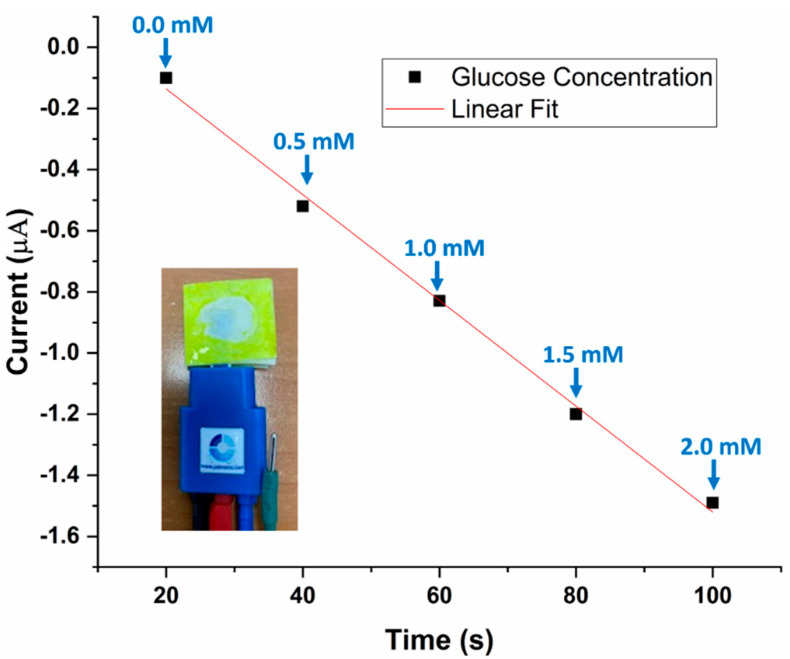
The linearity of the amperometric response of the glucose sensor at −0.1 V (w.r.t the Ag/AgCl R.E) at different glucose concentrations (0 mM~2.0 mM, in 0.01 M PBS solution), added in increments of 0.5 mM at intervals of 20 s. Inset: Experimental setup with the glucose sensor.

Moreover, the resulting amperometric response was found to vary linearly with time (Figure 7). The results of this experiment confirm that the 3D PMED developed in this study can measure varying glucose concentrations (in simulated sweat) in real-time, at relatively short intervals of time (20 s). Figure 6 displays the amperometric current response at 0.1 V (vs. Ag/AgCl R.E.) with alternate injecting of glucose (0.5 mM~2.0 mM glucose in increments of 0.5 mM) and PBS. In order to increase the evaporation rate and flowing of fluid, the sweat evaporator was covered with a block filter paper. The successive injecting was carried out at intervals of 500 s to allow the fluid refresh effectively (Figure 6).

### 3.4. SPE Selectivity for Glucose

**Figure 8:**
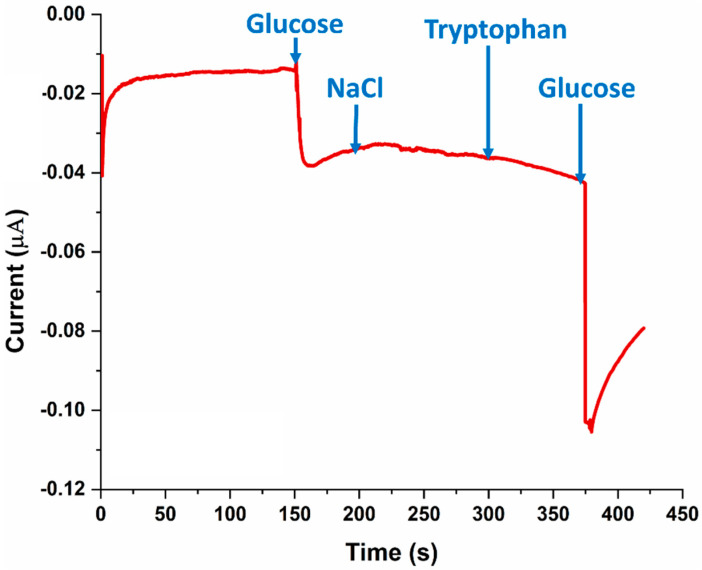
Demonstrating device selectivity towards glucose experiment measuring the amperometric current responses of the electrochemical biosensor with successive additions of glucose (solution in 0.01 M PBS), NaCl, tryptophan, and glucose again, at an applied potential of −0.1 V.

In order to confirm that the amperometric current response noted in Figure 5 and Figure 8 was specific to glucose, a selectivity experiment was performed. Briefly, glucose, sodium chloride (NaCl), tryptophan, and glucose (again) were added in succession onto the SPE at an applied potential of −0.1 V. As can be seen in Figure 5, the amperometric current response was observed only when glucose was added to the SPE, while electroactive interfering chemical species such as NaCl and tryptophan did not yield a current change.

### 3.5. On-Body Sweat Glucose Measurements

**Figure 9:**
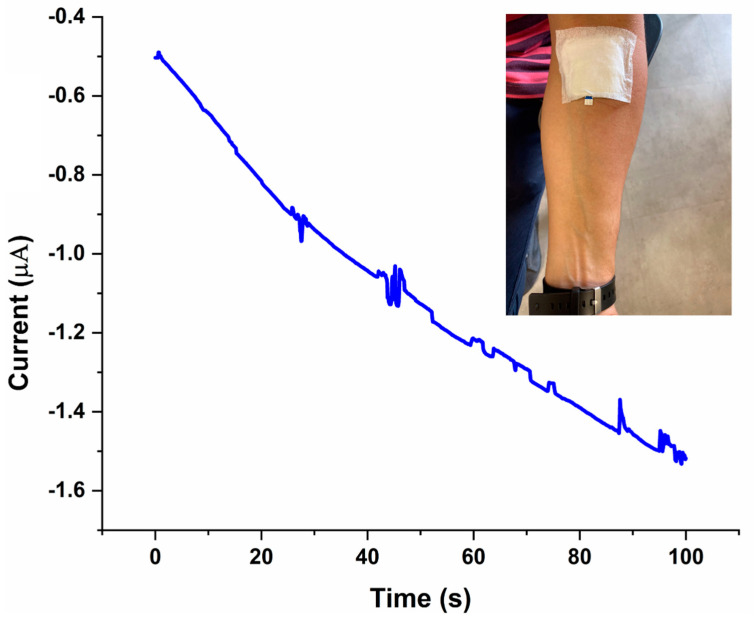
On-body measurement of glucose in sweat with the 3D PMED.

After confirming its functionality off-body, the 3D PMED was used for on-body glucose measurements in sweat. The device was placed on the study participant’s forearm (Figure 9), one of the most easily accessible sites in the body where the sweat glucose-secreting eccrine glands are present. Before exercise, the subject was asked to drink 200 mL honey water (honey:water ¼ 1:19, volume ratio) to raise their blood glucose. After digesting for 10 min, the participant was asked to perform a mild cycling exercise while the amperometric response of the device to sweat glucose was recorded (Figure 9). The current response increased with time, corresponding to an increase in the amount of sweat, with fluctuation ranging from 1.5 mM to 0.4 mM (and sweat glucose) available on the surface (Figure 8).

## 4. Discussion and Conclusions

The present work was a proof-of-concept study wherein a 3D PMED was developed to determine the glucose concentration in sweat, and its application was demonstrated on a human participant.

The 3D PMED glucose sensor developed in the present study was found to be selective for glucose. Note that sweat is primarily composed of water (99%), while the remaining 1% of the sweat is a mixture of ions (both metal and non-metal ions, such as sodium, potassium, and chloride ions), micronutrients (such as vitamins, iron, zinc, magnesium, etc.), amino acids (such as histidine), glucose, urea, ammonia, lactate, pyruvate, and other metabolites (including drug metabolites) [8,17,18]. In the present study, NaCl and tryptophan were selected for glucose selectivity testing of the 3D PMED because both NaCl and tryptophan are electroactive molecules belonging to different classes of compounds (electrolytes and amino acids, respectively) found in sweat [8,19]. Indeed, tryptophan is an electroactive amino acid that has extensively been explored as an analyte in the development of numerous electrochemical sensors [17]. Future studies could include other interfering electroactive molecules, such as lactate, pyruvate, and urea, to further confirm the device’s selectivity to glucose [11].

The primary reason for the glucose selectivity observed in the present study was the use of the NEXAR™ coating. Some previous studies on sweat metabolite analysis used a Nafion membrane instead of NEXAR™ to separate critical interfering electroactive molecules in sweat (such as chloride ions) from the absorbed sweat samples [11,20]. Nafion^®^-based devices have also shown similarly high selectivity towards glucose [11,21]. It should, however, be noted that some studies fail to test glucose selectivity in the presence of sodium chloride [21], the most prevalent interfering substance in sweat. Moreover, NEXAR™ polymer, which is a sulfonated pentablock copolymer, has greater electrolyte separation capacity than Nafion [22]. This is why the present study employed a NEXAR™ coating instead of Nafion.

NEXAR™ was previously used for desalination, oil separation, water filtration, and antimicrobial applications [22,23]. However, to the best of our knowledge, this is the first time NEXAR™ has been used for biosensing applications. Pertinent to its applicability in sweat glucose sensing, NEXAR™ has a higher electrolyte separation capacity compared to most other membranes commonly used for electrolyte separation. For example, NEXAR™-based membranes have been reported to have a water–ethanol separation factor of 127, which is higher than most other membranes (with separation factors lower than 100) [22]. Furthermore, owing to its higher acidity, NEXAR™ absorbs more water than Nafion^®^, making it more suitable for sweat-based biosensing applications. As a result of its greater water uptake capacity, NEXAR™ also has a significantly higher conductivity (2.63–4.84 mS/cm) than Nafion^®^ (0.645–0.703 mS/cm) [24].

It is important to note that glucose concentration in sweat (some reports suggest 0.25–1.5 mM as the range [11], others suggest 0.01–1.11 mM [2]) is significantly less than in blood (2–40 mM) [2]. However, sweat glucose is significantly correlated with blood glucose, even in diabetes patients, which makes it an important analyte for continuous, non-invasive glucose testing in diabetics [25]. Nevertheless, the smaller glucose concentrations in sweat make the development of an efficient sweat-based glucose sensor challenging because of the need for high sensitivity [2,11]. The glucose sensor developed in the present study had an acceptable sensitivity of 16.8 µA/mM/cm^2^, and the device was able to measure glucose in the 0–2 mM range, compatible with the range observed for sweat glucose. Future investigations should also include the evaluation of the device’s limit of detection by successively adding increasing amounts of glucose and registering the amperometric response yielded by the device. The smallest amount that is able to achieve a discernible amperometric response will be the limit of detection.

The final part of this study successfully demonstrated the ability of the glucose-sensing 3D PMED to detect sweat glucose in an on-body setting. Off-body tests, that is, measurements of sweat glucose concentrations using collected samples away from the body in a laboratory setting, are infeasible since they pose challenges of evaporation (and thus varying concentrations), degradation of chemicals between collection and analysis, inability or difficulty to collect the required amount of sweat sample (preferably greater than 10 µL), and the need for expensive, voluminous instrumentation [2]. Wearable sweat-based glucose sensors, such as the one developed in the present study, overcome these challenges by offering non-invasive, autonomic, continuous, and real-time sample collection and analysis [2].

One of the limitations of the present study is the use of a single study participant. To extend the results of this study beyond its current proof-of-concept stage, it is necessary to include a greater and statistically relevant number of study participants. It is also important to test the developed 3D PMED under different exercise regimens and in human participants with different skin conditions. Moreover, the sweat collection site of the 3D PMED should also be altered to test if eccrine gland sites other than the forearm (such as the forehead, upper back, mid-back, lower back, etc.) are better suited for sweat glucose quantitation. This is because the sweat rate for an individual depends not only on the intensity of the exercise regimen but also on the location of the body [26,27]. In a study conducted on male athletes, it was reported that at a less intense exercise regimen (55% maximal oxygen uptake), the mid-back has the highest sweat rate (771 g/m^2^/h), while at a higher intensity exercise regimen (75% maximal oxygen uptake), the forehead is the site of the highest sweat rate (1710 g/m^2^/h) [27]. The applicability of this device in diabetes patients would require extensive testing with diabetic study participants and the establishment of a one-to-one correlation of the sweat glucose concentrations with the corresponding blood glucose concentrations.

The present study demonstrates the development and characterization of a 3D paper-based microfluidic electrochemical integrated device (3D-PMED) for measuring glucose concentration in sweat in real-time via simple, non-invasive, capillary-action-based sample collection. The device was selective for glucose and was able to detect glucose in the clinically relevant range of 0–2 mM in an off-body setup. Further, the developed glucose sensor had an acceptable sensitivity of 16.8 µA/mM/cm^2^.

On-body testing has been performed in many different ways in previously reported studies. For example, in one study, the sensor was sewn onto an arm guard, and the study participants were asked to run for 25 min before on-body sweat glucose levels were recorded [28]. Another study applied the sensor on the participants’ foreheads as they performed a cycling exercise [29]. A further study applied the sensor on the participant’s forearm while they performed a 25 min cycling exercise [11]. The method used for on-body sweat glucose measurements in the present study was comparable to previous reports. Specifically, on-body glucose measurements were obtained by placing the device directly on the forearm of the participant while they performed a cycling exercise. In the on-body setup, the device was able to achieve a significant amperometric response to sweat glucose in a very short amount of time (a few seconds). With detailed investigations, this proof-of-concept study could help further the development of sensitive and selective sweat-based glucose sensing devices for real-time glucose monitoring in diabetes patients.

## Figures and Tables

**Table 1:** Amperometric response to successively increasing glucose concentrations to determine the sensitivity of the 3D PMED glucose sensor.

Time (s)	Glucose Concentration (mM)	Current (µA)
20	0.0	−0.10
40	0.5	−0.52
60	1.0	−0.83
80	1.5	−1.20
100	2.0	−1.49

## Data Availability

All relevant data are available in the manuscript.
